# Spatial Analysis of Land Cover Determinants of Malaria Incidence in the Ashanti Region, Ghana

**DOI:** 10.1371/journal.pone.0017905

**Published:** 2011-03-23

**Authors:** Anne Caroline Krefis, Norbert Georg Schwarz, Bernard Nkrumah, Samuel Acquah, Wibke Loag, Jens Oldeland, Nimako Sarpong, Yaw Adu-Sarkodie, Ulrich Ranft, Jürgen May

**Affiliations:** 1 Infectious Disease Epidemiology, Bernhard Nocht Institute for Tropical Medicine, Hamburg, Germany; 2 IUF - Leibniz Research Institute for Environmental Medicine, Heinrich Heine University Düsseldorf, Düsseldorf, Germany; 3 Kumasi Centre for Collaborative Research in Tropical Medicine, Kumasi, Ghana; 4 School of Medical Sciences, Kwame Nkrumah University of Science and Technology, Kumasi, Ghana; 5 Biocentre Klein Flottbek and Botanical Garden, University of Hamburg, Hamburg, Germany; Indiana University at Bloomington, United States of America

## Abstract

Malaria belongs to the infectious diseases with the highest morbidity and mortality worldwide. As a vector-borne disease malaria distribution is strongly influenced by environmental factors. The aim of this study was to investigate the association between malaria risk and different land cover classes by using high-resolution multispectral Ikonos images and Poisson regression analyses. The association of malaria incidence with land cover around 12 villages in the Ashanti Region, Ghana, was assessed in 1,988 children <15 years of age. The median malaria incidence was 85.7 per 1,000 inhabitants and year (range 28.4–272.7). Swampy areas and banana/plantain production in the proximity of villages were strong predictors of a high malaria incidence. An increase of 10% of swampy area coverage in the 2 km radius around a village led to a 43% higher incidence (relative risk [RR] = 1.43, p<0.001). Each 10% increase of area with banana/plantain production around a village tripled the risk for malaria (RR = 3.25, p<0.001). An increase in forested area of 10% was associated with a 47% decrease of malaria incidence (RR = 0.53, p = 0.029).

Distinct cultivation in the proximity of homesteads was associated with childhood malaria in a rural area in Ghana. The analyses demonstrate the usefulness of satellite images for the prediction of malaria endemicity. Thus, planning and monitoring of malaria control measures should be assisted by models based on geographic information systems.

## Introduction

With 250 million estimated malaria cases in 2008 and one million deaths malaria is the most common vector-borne infectious disease with Sub-Saharan Africa carrying most of the burden. In regions of stable transmission children <5 years of age are at highest risk of becoming symptomatic after infection with malaria parasites. The causal protozoon *Plasmodium falciparum* is transmitted from person to person through the bite of adult female *Anopheles* mosquitoes [1], [2].

Transmission and prevalence of vector-borne diseases such as malaria are highly influenced by spatial and temporal changes in the environment as described during the last 20 years by geographic information systems (GIS) and remotely sensed (RS) data [3], [4]. Studies mapping potential mosquito habitats, transmission risk, or disease prevalence have been performed in Africa [5]–[8], South and Central America [9], [10], and Asia [11], [12]. However, analyses of the direct correlation between environment and malaria are rare.

Adult vector abundance is positively associated with the availability of aquatic habitats necessary for the deposition of eggs, and the areas with highest malaria risk are often found within just a few hundred meters of such larval habitats [13], [14]. It has been suggested that extensive cultivation of maize might influence the larval development of mosquitoes, pupation success, and size of adults in the vicinity [15]. Recent studies from Kenya have shown that highland habitats created by deforestation or cultivation of natural swamps were associated with preferred breeding habitats [16], [17].

In Ghana, where the present study was conducted, malaria is prevalent during the entire year and accounts for about 32–42% of all outpatient admissions and for the major in-patient causes of death [18]. The main malaria vectors are mosquitoes of the *Anopheles gambiae* complex and *A. funestus* [19].

The aim of the study was to investigate the association between malaria incidence and different classes of land cover that potentially influence the malaria vector abundance as well as human population density. High spatial resolution satellite images as well as statistical modeling was used to assess the influence of land cover classes and the human population at risk on the malaria incidence (per year and 1,000 inhabitants) in children <15 years of age in an area of high endemicity. This information might be of importance to the understanding of environmental determinants of malaria transmission heterogeneity at a micro-geographical scale.

## Materials and Methods

### Ethics Statement

Aims and principles of the study were explained in detail to participants and informed consent was obtained by signature or thumb print by the caregiver. The study design and the informed consent form were approved by the Committee on Human Research, Publications, and Ethnics, School of Medical Sciences, Kwame Nkrumah University of Science and Technology, Kumasi, Ghana.

### Study area and data collection

**Figure 1:**
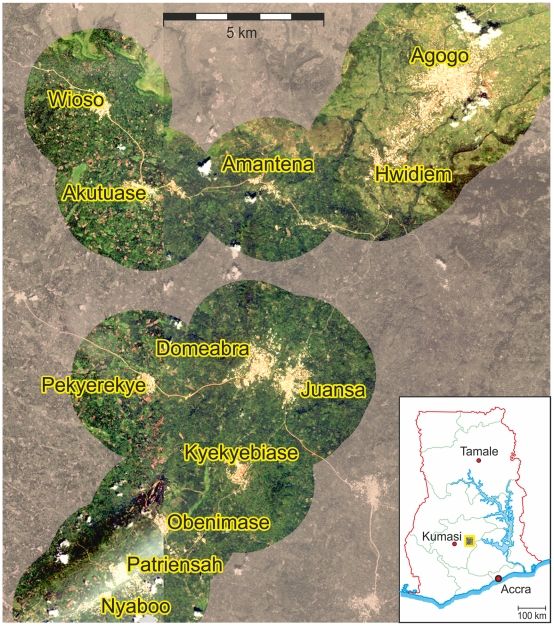
Map of the 12 included study villages. Merger of two satellite images (Ikonos) depicting an area with 12 study villages in the Asante Akim North District, Ashanti Region, central Ghana, West Africa. Areas with a radius of 2 km surrounding the study villages, which were analysed by supervised maximum likelihood classification, are coloured.

The hospital-based survey was accomplished at the Child Welfare Clinic and the Pediatric Ward of the Agogo Presbyterian Hospital (APH), Asante Akim North District, Ashanti Region, Ghana. The study area was restricted to the 12 study villages Agogo, Hwidiem, Akutuase, Amantena, Wioso, Domeabra, Juansa, Kyekyebiase, Nyaboo, Obenimase, Patriensah and Pekyerekye and their 2 km surrounding areas (Figure 1). The total area of our study side covered approximately 170 km^2^. For more detailed information about the study area see Krefis *et al.* 2010 [20].

All children <15 years of age visiting the Child Welfare Clinic of the APH and with permanent residency in one the 12 study villages were examined for malaria (criterion: fever ≥37.5°C and positive for asexual *P. falciparum* parasitaemia with >0 parasites/µL) during the study interval of 18 months (end of May 2007 to November 2008). Parasite examination was done according to quality-controlled standardised procedures described elsewhere [21]. Malaria cases presented within 21 days after the initial malaria diagnosis were considered as a relapse and not counted as a new case.

For the calculation of incidences the population size, the admission rate, the proportion recruited, and the proportion of the population seeking health care in the study hospital were considered. Population figures were taken from the population census 2004 with the estimate that 42% of the individuals were <15 years of age [22]. The proportion of people from each village attending the APH was assessed by a community survey on health seeking behaviour that was carried out in 2007 and the denominator/reference population for the calculation of incidence was corrected for these proportions [23]. After comparing the study population with the hospital admission records it was estimated that 70% of all individuals (range: 67% to 72%) admitted to the hospital were included on average into the study and therefore the reference population was likewise corrected for this factor. Finally, annual malaria incidences per 1,000 children <15 years of age were computed for each of the villages. The human population density per village was computed by using population census and village area data (Table 1).

**Table 1:** Characteristics of the villages.

Village	Village area^a^	Total population^b^	Population density^a^	Proportion with hospital access^c^	Population study group^d^	Malaria cases^e^	Incidence^f^
Agogo	5.12	13559	2648	90%	3588	1463	271.9
Akutuase	0.61	1692	2774	43%	214	9	28.1
Amantena	0.27	890	3296	55%	144	21	97.3
Domeabra	1.33	3509	2638	42%	433	73	112.3
Hwidiem	1.08	1402	1298	95%	392	147	250.3
Juansa	1.27	3992	3143	40%	469	52	73.8
Kyekyebiase	0.54	1801	3335	46%	244	28	76.6
Nyaboo	1.02	1582	1551	46%	214	28	87.2
Obenimase	0.51	1096	2149	37%	119	15	83.9
Patriensah	0.54	4463	8265	38%	499	92	123.0
Pekyerekye	0.37	1692	4573	45%	224	27	80.4
Wioso	0.34	1783	5244	52%	273	33	80.7

a per km^2^.

b Population according to the national census 2004 [21].

c Proportion of people in each village who reported to visit the Agogo hospital, data from Community Survey.

d Population of children <15 years of age estimated as a proportion of 42% of the population counted at national census 2004 [22] and an additional proportion of 70% due to inclusion into the study at hospital admittance and by taking into account hospital access.

e Study period from May 2007 to November 2008 (18 months).

f Incidence in children <15 years (per year and 1,000 children <15 years).

Formula: incidence = 1,000*cases/(total_population*0.42*0.70*hospital_access*1.5).

### Mapping land cover classes using remote sensing

In order to map the land cover classes around each village, we acquired two multispectral Ikonos images with 4-meter spatial resolution and four broad spectral bands (wavelengths: blue, 0.45–0.52 µm; green, 0.52–0.59 µm; red, 0.62–0.68 µm; and near-infrared (NIR), 0.77–0.86 µm), along with one panchromatic band with 1 pixel/m. Images were acquired on May 4, 2009, and on November 26, 2009 (it was not possible to obtain two contemporaneous images of high quality from the area due to weather, cloud condition, and other acquisition difficulties).

All pre-processing steps were carried out using ENVI 4.4 (ITTVIS, 2009). For easier computation the images were divided into subsets, each covering one or two village areas. For each subset a Normalized Difference Vegetation Index (NDVI = [NIR−red]/[NIR+red]) image was calculated, which is a commonly used measure of vegetation productivity [24]. Beside the spectral domain, the spatial domain was also considered by calculating a set of textural measures based on a grey level co-occurrence matrix in order to improve the classification [25], [26]. Different textural measures (contrast, homogeneity, angular second moment, variance, mean, dissimilarity, entropy, and correlation) were received by moving several windows of different pixel areas (3×3 to 15×15) over the image, leading to a new textural image for each measure. The optimal window size was determined by using a confusion matrix to assess the accuracy of the solely texture based classification [25]. Afterwards, the textural images were combined with the NDVI image and the four spectral bands for further analysis.

In March 2010, field sampling of different land cover classes was conducted by the direct inspection of 490 points randomly selected in the vicinity of the 12 study villages. We marked the points using a Garmin eTrex®H Global Positioning System (GPS) and took notes and photographs on the dominant vegetation or crop type.

By using the ENVI software, these reference areas were digitised as regions of interest and were used to represent one of the following land cover classes: banana or plantain, cacao, palm trees, oranges, swampy area, water, deforested area and roads, built-up areas (houses), and forest. Classes describing the crops “banana/plantain”, “cacao”, “palm trees” producing palm oil fruits, and “oranges” were mostly mixed fields but dominated by one of these crops, respectively. Either the presence of a river or stream nearby or near the ground agricultural crops (such as eggplants, maize, tomatoes, pepper), which was mostly cultivated in the vicinity, characterised the combined variable “swampy area”. “Water” was characterised by a river, stream or lake. “Deforested area” was characterised by burned, grassy or bushy underground or open spaces; additionally we assigned roads within this class. “Forest” referred to areas with dense tree cover with a closed canopy.

It was not possible to get images completely free of clouds. Therefore, two additional classes, one for clouds and one for the shadow of a cloud were generated to mask out those particular areas.

All combined bands were classified using a supervised maximum likelihood classifier. Therefore, a random subset of 70% of the pixels for each of the classes was chosen for a basic analysis (“training data”) and 30% were used for assessment of accuracy (“validation data”). In the post classification process, we applied a majority/minority analysis for generalisation of the classification image to minimise “salt and pepper effects”, a term which describes the existence of dark pixels in bright regions and bright pixels in dark regions, usually causing noise in the validation procedure. Validation of the accuracy of the post-processed classification image was based on the overall accuracy computed from the confusion matrix. The maximum likelihood classification is usually considered to be satisfiable when the overall accuracy is higher than 85%. The final image was transferred to ArcGIS version 9.3, developed by Environmental System Research Institute (ESRI, 2008).

**Figure 2:**
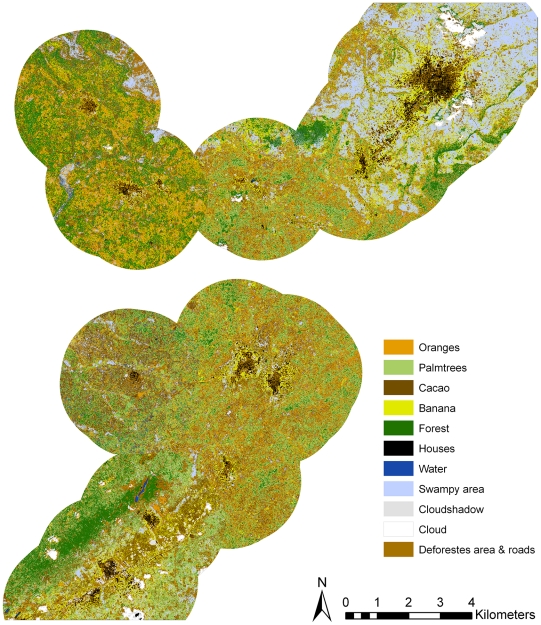
Supervised maximum likelihood classification map (combined NDVI image, the texture bands, and the four spectral bands). Classification of land cover within a village radius of 2 km with 11 colours indicating different land cover classes. All classes describing the crops banana/plantain, cacao, palm trees producing palm oil fruits, and oranges were mainly mixed fields but dominated by one of these crops. Swampy areas were characterised by either the presence of a river or stream nearby or an agricultural crop cultivated in the vicinity. Water was characterized by a river, stream or lake. Deforested areas were characterized by roads as well as burned, grassy or bushy underground or open spaces. Forest referred to areas with dense tree cover, mostly with a closed canopy.

Taking into account that adult mosquitoes remain generally up to 2 km of their breeding side [27]–[29] a radius of 2 km around each village was created and the percentage of various land cover classes in each radius was computed. Due to the particular size of the village Agogo an oval-shaped radius was used (Figure 2). In order to test the validity of the analyses additional radii of 0.5 km, 1 km, and 1.5 km around each village were used.

### Analyses and statistical modeling

The quantitative assessment of associations between proportional land cover and the incidence of malaria was done by Poisson regression analyses with adjustment for overdispersion (STATA/SE software, version 10; Stata Corp LP, College Station, TX). By using Spearman rank correlation we calculated the cross correlation between potential determinants for malaria: population density as a measure for human-mosquito-contact, deforested area and roads, swampy area, respectively prone to the formation of puddles and hence breeding sites, water, houses to look for shelter for mosquitoes during daytime, forest, and vegetation of banana/plantain, oranges, cacao, and palm trees as potential resting and breeding sites or food sources. Land cover proportions were analysed as continuous variables and human population density as per 1,000 inhabitants. The approximated interquartile range was used as unit increase for the continuous variables.

Because of the small sample size (12 village clusters), the influence of each potential determinant was assessed separately in a univariate Poisson regression in a first step. For a measure of association between a determinant and malaria incidence, the relative risk (RR) was calculated and complemented by a 95% confidence interval (CI) and p-value. In a second step as sensitivity analysis and to account for confounding, the high correlated determinants with a p-value less than 0.05 were included in a bivariate Poisson regression analysis.

## Results

### Malaria incidence and human population density

A total of 1,988 malaria cases were reported in the study hospital during the study interval of 18 months (end of May 2007 to November 2008) and were included in the analysis. Annual malaria incidence ranged from 28.1 to 271.9 per 1,000 children <15 years of age and year in Akutuase and Agogo, respectively. A crude annual malaria incidence of 194.5 per 1,000 children <15 years age and year with a 95% confidence interval of [144.9, 261.3] could be estimated for the whole study area. The total population of the study villages was 37,461 inhabitants (census data 2004). The human village population density ranged from 1,298 inhabitants/km^2^ in Hwidiem to 8,265 inhabitants/km^2^ in Patriensah (Table 1).

### Classification and correlation of land cover determinants

All four broad spectral bands (blue, green, red, and near-infrared) from the acquired multispectral Ikonos image along with the NDVI image were considered in our study. A window size of 9×9 pixels (equivalent to 9×9 m) had the highest accuracy of the texture-based classification and respective textural measures were chosen for the analyses accordingly (data not shown).

By using reference areas for all nine land cover classes a maximum likelihood classification was conducted of the combined NDVI image, the spectral, and the textural bands. Overall accuracy of the classification ranged from 87% in Wioso and Akutuase to 95% in Obenimase.

The proportion of areas with banana/plantain vegetation within a village radius of 2 km varied from 4.8% in Pekyerekye to 19.7% in Agogo. The highest proportion of swampy area was found around the village Agogo (37.0%), the lowest proportions (4.7% and 4.9%) around the two villages Akutuase and Juansa, respectively. The proportion of forest coverage varied from 6.3% around Domeabra to 28.7% around Wioso (Table 2, Figure 2).

**Table 2:** Proportion (in %) of land cover around a 2 km village centre radius.

Village radius^a^	Banana/Plantain	Cacao	Palm trees	Oranges	Deforested area and roads	Built-up areas (Houses)	Swampy area^b^	Water	Forest
Agogo	19.7	9.5	4.5	6.9	9.2	4.1	37.0	0.1	6.4
Akutuase	11.5	10.9	4.1	28.7	9.4	1.4	4.7	1.4	27.9
Amantena	12.1	22.2	23.3	20.4	2.6	0.7	7.5	0.1	10.1
Domeabra	9.8	26.2	23.9	22.7	3.9	2.1	5.0	0.0	6.3
Hwidiem	18.7	13.3	12.6	13.2	5.6	2.6	23.9	0.1	9.9
Juansa	9.2	25.9	24.5	24.0	3.8	2.2	4.9	0.0	5.5
Kyekyebiase	7.3	28.9	24.3	26.6	1.6	0.6	5.2	0.1	5.4
Nyaboo	17.4	15.5	13.9	2.2	19.6	3.9	8.9	0.1	15.1
Obenimase	14.0	19.2	19.5	3.0	17.6	2.5	6.9	0.3	15.0
Patriensah	16.2	15.1	16.5	2.3	18.4	3.4	8.9	0.0	16.0
Pekyerekye	4.8	26.4	22.1	13.5	9.1	0.6	8.4	4.3	10.8
Wioso	9.3	14.4	5.0	22.5	10.2	0.7	7.8	1.2	28.7

a Size of each radius 2 km^2^.

b Swampy area: either the presence of a river or stream nearby or near the ground agricultural crops (such as eggplants, maize, tomatoes, pepper).

Spearman rank tests resulted in high positive correlations between the land cover proportions of forest and deforested area/roads (r = 0.79, p = 0.002), banana/plantain and built-up areas (r = 0.86, p<0.001) as well as palm trees and cacao (r = 0.91, p<0.001) (Table 3). Highest negative correlations were observed between the land cover proportions of cacao and banana/plantain (r = −0.73, p = 0.007) and swampy area and oranges (r = −0.78, p = 0.003).

**Table 3:** Correlation coefficients of the cross correlation function between land cover variables using Spearman rank correlation.

	Population density	Built-up areas (Houses)	Deforested area and roads	Forest	Swampy area	Water	Banana/Plantain	Oranges	Cacao	Palm trees
Population density		−0.51	−0.08	0.22	−0.11	0.07	−0.55	−0.24	0.24	0.17
Built-up areas (Houses)	0.09		0.55	0.08	0.52	−0.38	0.86	−0.66	−0.62	−0.41
Deforested area and roads	0.80	0.06		0.79	0.37	0.23	0.46	−0.66	−0.52	−0.62
Forest	0.50	0.81	0.002		0.14	0.42	0.18	−0.30	−0.51	−0.67
Swampy area	0.75	0.08	0.23	0.66		−0.03	0.64	−0.78	−0.42	−0.43
Water	0.84	0.22	0.48	0.17	0.92		−0.24	0.13	−0.14	−0.46
Banana/Plantain	0.06	<0.001	0.13	0.57	0.02	0.46		−0.67	−0.73	−0.57
Oranges	0.46	0.02	0.02	0.34	0.003	0.70	0.02		0.27	0.26
Cacao	0.44	0.03	0.08	0.09	0.17	0.66	0.007	0.39		0.91
Palm trees	0.60	0.19	0.03	0.02	0.16	0.13	0.05	0.42	<0.001	

Note: Above the diagonal are the correlation coefficients r, below all p-values.

As expected, the proportions of land cover in the vicinity of villages in the 0.5 km, 1 km, and 1.5 km radii were not exactly the same from what was found in the 2 km village radius. The proportions of built-up areas (houses), deforested areas and roads, and banana/plantain vegetation decreased with distance to the village whereas the proportion of areas with forest, palm trees, orange trees, and cacao trees increased (Tables S1, S2, S3).

### Regression modeling

In the univariate Poisson regression analysis, all determinants with the exception of population density, water, and deforested area and road coverage showed a significant influence on malaria incidence, which was positive for banana/plantain cultivation (RR = 3.25), swampy areas (RR = 1.43), and built-up areas (RR = 2.24), but negative for forest (RR = 0.53), orange (RR = 0.63), cacao (RR = 0.48), and palm trees (RR = 0.59) plantation (Table 4). However, in the sensitivity analysis by means of bivariate Poisson regression analysis, the univariate results for built-up areas and orange, cacao, and palm tree plantation turned out to be confounded because of high correlation between several determinants (Table 3) and not to be of statistical significance (data not shown).

**Table 4:** Influence of determinants on malaria incidence^a^.

Determinant	RR	95% Confidence Interval	p-value
Population density^b^	0.87	0.70–1.07	0.176
Built-up areas (houses)^c^	2.24	1.54–3.24	<0.001
Deforested area and roads^d^	1.00	0.70–1.44	0.988
Forest^e^	0.53	0.28–0.99	0.029
Swampy area^e^	1.43	1.33–1.55	<0.001
Water^f^	0.70	0.37–1.32	0.270
Banana/Plantain^e^	3.25	2.23–4.76	<0.001
Oranges^e^	0.63	0.44–0.91	0.012
Cacao^e^	0.48	0.33–0.70	<0.001
Palm trees^e^	0.59	0.43–0.81	<0.001

a Poisson regression analysis.

b Unit: 1,000/km^2^.

c Unit = 2%.

d Unit = 5%.

e Unit = 10%.

f Unit = 1%.

The association of land cover with malaria incidence in the other 3 radii were similar to those of the 2 km radius (Table S4) and the sensitivity analysis showed similar results (data not shown).

## Discussion

The risk for malaria is dependent on a number of individual and environmental factors whereas their impact is dependent on the endemicity in a certain area [15], [16], [20]. Recently, it has been shown that the spatial variance of malaria incidence might be pronounced not only in areas with low and seasonal endemicity but also in holoendemic areas [20], [30]. It can be assumed that mosquito occurrence, the existence of breeding sites, and human population density are the most important spatial determinants, all significantly linked to land cover and land use [8], [13], [14], [30]. Accordingly, land cover has been associated with entomological measures mainly accumulated as entomological inoculation rates (EIR, infectious mosquito bites per person per year) [3], [5]. Studies using high-resolution satellite images in association with vector-borne diseases have already been conducted in other areas [3]–[12]. However, analyses of the direct relationship between environmental factors and human malaria, especially using high-resolution images and/or subclassification of land cover in such detail are scarce, most probably due to the absence of precise data on malaria incidence and exact description of the land cover in large areas.

The presented analyses have used malaria incidence data over 18 months from a hospital-based survey and high-resolution satellite images of a holoendemic coverage area in the Ashanti Region, Ghana. The analyses demonstrate that an accurate stratification of land cover by satellite images is possible in areas of small-scale cultivation and changing agriculture. Land cover of banana/plantain vegetation and swampy areas significantly increased the malaria risk. In contrast, an increased proportion of forest around villages was associated with decreased malaria risk. These trends remained when conducting the analysis using smaller radii. However, the RRs for banana/plantain cultivation and forest vegetation decreased with each diminished radius. This trend may be explained by the increasing proportions of banana vegetation and decreasing proportions of forest near homesteads and hence the reducing divergence among all villages (Tables S1, S2, S3).

The increase of malaria risk in the vicinity of swampy areas, which are preferred mosquito breeding sites, has already been documented and can be considered as an internal control of the validity of the analyses [13], [14], [16], [31]. In the presented model each 10% increase of the proportion of swampy areas around villages increased the malaria risk by 43%.

A higher proportion of all cultivated areas around villages was associated with a slightly increased risk of malaria (data not shown). The main plantations in the study area are with banana/plantains, oranges, cacao, and palm trees [32]. After stratification for the distinct cultivations, a plantation with banana/plantain was found of particular impact and a 10% increase was associated with about 300% higher malaria risk whereas plantations of oranges, cacao, and palm trees showed a negative association. A number of studies that were conducted at a microhabitat scale demonstrated an association between ovipositions of various mosquito species in rainwater retained in tree-holes and the leaf axils of a variety of numerous wild and cultivated plants such as banana or plantain [33]–[36]. However, none of these studies has directly linked the existence of breeding sites with malaria incidence.

Likewise, deforested areas and dirt roads have been suspected as environmental factors associated with malaria risk in the surrounding areas since both create conditions favourable for the formation of small puddles that are preferred breeding sites for *Anopheles spp.* [16], [17], [29], [37],[38]. However, a significant influence of deforested areas and roads on malaria incidence could not be observed in the presented study. Similarly, population density as a measure for potential human-mosquito contact did not show an effect on the outcome.

A high proportion of forest coverage was associated with lower malaria incidence with statistical significance. Indeed, the forest floor with a closed canopy tends to be heavily shaded and littered with a thick layer of organic matter that absorbs water and renders it more acidic. Therefore, the proximity of forest could decrease mosquito abundance and hence decrease malaria risk as the preferred habitat of *A. gambiae* larvae are sunlit pools with turbid water and little or no emergent vegetation and that of *A. funestus* are clear water with vertical, emergent vegetation without organic material [31], [37].

There was a tendency of an association between an increased proportion of build-up areas and malaria incidence. However, this effect disappeared after adjustment for the highly correlated variable “banana/plantain” what indicates confounding which is, however, difficult to formally test due to the ecological study design. The observation that vicinity to banana/plantain cultivations seems to be a risk factor for malaria may be because of the frequent closeness of this vegetation with homesteads.

A limitation of our study is that the proportion of children <15 years of age in each village was estimated and could not directly be measured when computing malaria incidences. Moreover, the underlying values for the total population were three years old (census data from 2004) and hence might be not precise. However, it was the best data available for our study population and census data not more than five years old might represent a quite good estimate. Additionally, the villages should be comparable in the proportions of children since they have similar social and ethnic structures, are of similar size, and are all situated in a rural area and closely together. Therefore, it is unlikely that a differential bias was created.

Climate conditions are suspected to be of importance for the malaria risk and higher precipitation could be directly linked, with a time lag, to an abundance of vectors and an increase of disease frequency [39]. The satellite images, which were analysed here, were taken during or immediately after the rainy season (May and November). Therefore, most of the open lakes, rivers or streams were most likely detected and included in our analysis. Nevertheless, an association of the proportion of open water bodies and risk of malaria could not be demonstrated in the presented study. A limitation of the analysis is that the proportion of open water in the surrounding of the villages was very low and streams and little rivers were mostly located close to forests and, therefore, difficult to detect on our satellite images.

Another limitation of the analyses was that some areas were covered with clouds and their shadows in both images. However, the proportion of cloudy areas was very low (<5% in total) and randomly distributed hence should not significantly affect the results.

In Ghana, the major farming practice is shifting cultivation, often accompanied by deforestation, and crops mostly change twice to three times a year [32]. Due to the fact that a time span of nine and four months occurred between the acquisition of images (May and November 2009) and the conducted field sampling (March 2010), respectively, the assigned land cover might be biased. By interviewing the local population about previous crops and land cover we attempted to minimise this potential misclassification.

Maximum likelihood procedures were used for supervised classification of land cover data, which gave more accurate results than other classification methods such as Decision Tree-, Minimum Distance- or K-Means Classification [40]–[42]. Even though the overall accuracy of the correlation matrix of the NDVI image, the spectral and the spatial classification in the subsets ranged from 87% to 95%, land cover still might be misclassified to some extent. In the study area as in most areas of Ghana mixed cultivation is widespread [32] which makes it very difficult to unambiguously allocate land covers.

Our study was limited by the inability to sub-classify swampy areas, which are mostly used for near-ground cultivation, into different crops such as maize, eggplants or pepper for the analysis of various influences on malaria risk. In Ethiopia a strong association between maize cultivation in the vicinity of water bodies, used as breeding habitats, and the larvae development was demonstrated [15]. However, because of a high number of classes in relation to 12 village clusters and weak accuracy of data in this classification analyses, this sub-classification was not possible.

Other geo-ecological influence factors for the malaria risk are altitude, slope, geology, and soil types [29], [43]–[46]. However, the intra-radius variation in these measures did not differ significantly.

The consequent next step should be to map individual data in order to link individual spatial patterns and malaria risk. Indeed, in an adjacent study area a continuous and linear reduction of the malaria rate was demonstrated with an increasing distance between children's households and forest fringe [30]. Other individual factors such as socioeconomic conditions and the access to health facilities could then be included in the model [20], [47], [48].

The performed analysis demonstrates that satellite images together with appropriate analytical tools are able to predict the risk of malaria in an area of high malarial transmission. Even though only 12 village sides were included in the study a significant association of different land cover classes with the occurrence of malaria incidence could be demonstrated. Human cultivation in the vicinity of homesteads, in particular with banana/plantain, may increase the risk for malaria. On the contrary, forest preservation may decrease malaria risk. In the future, mapping of GPS positions of each household would enable to determine individual risk and to confirm and to improve the validity of the model. Malaria persists to be an important public health problem and policy makers should involve geographic information systems for planning and monitoring malaria control strategies.

## Supporting Information

Table S1 **Proportion (in %) of land cover around a 1.5 km village centre radius.** Swampy area: either the presence of a river or stream nearby or near the ground agricultural crops (such as eggplants, maize, tomatoes, pepper).
(DOC)

Table S2 **Proportion (in %) of land cover around a 1 km village centre radius.** Swampy area: either the presence of a river or stream nearby or near the ground agricultural crops (such as eggplants, maize, tomatoes, pepper).
(DOC)

Table S3 **Proportion (in %) of land cover around a 0.5 km village centre radius.** Swampy area: either the presence of a river or stream nearby or near the ground agricultural crops (such as eggplants, maize, tomatoes, pepper).
(DOC)

Table S4 **Influence of determinants on malaria incidence.** Association of land cover with malaria incidence using Poisson regression analysis in radii of 0.5 km, 1 km, 1.5 km, and 2 km around each village. Land cover proportions were analysed as continuous variables and were scaled by units as per 2% increase in radii coverage by built-up areas (houses), per increase in open water of 1%, per increase of deforested area and roads of 5%, per increase in forest, swampy area, banana/plantain, oranges, cacao, and palm tree vegetation of 10%, respectively, and human population density as per 1,000 inhabitants.
(DOC)

## References

[1] WHO: World malaria report. Geneva: World Health Organization (2009). http://apps.who.int/malaria/wmr2008/malaria2008.pdf.

[2] Hay SI, Okiro EA, Gething PW, Patil AP, Tatem AJ (2010). Estimating the global clinical burden of Plasmodium falciparum Malaria in 2007.. PLoS Med.

[3] Rogers DJ, Randolph SE, Snow RW, Hay SI (2002). Satellite imagery in the study and forecast of malaria.. Nature.

[4] Hay SI, Packer MJ, Rogers DJ (1997). The impact of remote sensing on the study and control of invertebrate intermediate hosts and vectors for disease.. Int J Remote Sens.

[5] Bogh C, Lindsay SW, Clarke SE, Dean A, Jawara M (2007). High spatial resolution mapping of malaria transmission risk in the Gambia, west Africa, using Landsat TM satellite imagery.. Am J Trop Med Hyg.

[6] Mushinzimana E, Munga S, Minakawa N, Li L, Feng CC (2006). Landscape determinants and remote sensing of anopheline mosquito larval habitats in the western Kenya highlands.. Malar J.

[7] Kulkarni MA, Desrochers RE, Kerr JT (2010). High Resolution Models of Malaria Vectors in Northern Tanzania: A New Capacity to Predict Malaria Risk?. PLoS ONE.

[8] de Souza D, Kelly-Hope L, Lawson B, Wilson M, Boakye D (2010). Environmental Factors Associated with the Distribution of *Anopheles gambiae* s.s in Ghana; an Important Vector of Lymphatic Filariasis and Malaria.. PLoS ONE.

[9] Roberts DR, Paris JF, Manguin S, Harbach RE, Woodruff R (1996). Predictions of malaria vector distribution in Belize based on multispectral satellite data.. Am J Trop Med Hyg.

[10] Beck LR, Rodriguez MH, Dister SW, Rodriguez AD, Rejmankova E (1994). Remote sensing as a landscape epidemiologic tool to identify villages at high-risk for malaria transmission.. Am J Trop Med Hyg.

[11] Sharma VP, Nagpal BN, Srivastava A, Adiga S, Manavalan P (1996). Estimation of larval production in Sanjay Lake and its surrounding ponds in Delhi, India using remote sensing technology.. Southeast Asian J Trop Med Public Health.

[12] Nihei N, Hashida Y, Kobayashi M, Ishii A (2002). Analysis of malaria endemic areas on the Indochina Peninsula using remote sensing.. Jpn J Infect Dis.

[13] Edillo FE, Toure YT, Lanzaro GC, Dolo G, Taylor CE (2002). Spatial and habitat distribution of *Anopheles gambiae* and *Anopheles arabiensis* (Diptera: Culicidae) in Banambani village, Mali.. J Med Entomol.

[14] Shililu J, Ghebremeskel T, Seulu F, Mengistu S, Fekadu H (2003). Larval habitat diversity and ecology of anopheline larvae in Eritrea.. J Med Entomol.

[15] Ye-Ebiyo Y, Pollack RJ, Spielman A (2000). Enhanced development in nature of larval *Anopheles arabiensis* mosquitoes feeding on maize pollen.. Am J Trop Med Hyg.

[16] Munga S, Minakawa N, Zhou G, Mushinzimana E, Barack OO (2006). Association between land cover and habitat productivity of malaria vectors in western Kenyan highlands.. Am J Trop Med Hyg.

[17] Minakawa N, Munga S, Atieli FK, Mushinzimana E, Zhou G (2005). Spatial distribution of anopheline larval habitats in western Kenya highlands: effects of land cover types and topography.. Am J Trop Med Hyg.

[18] De-Graft Aikins A (2007). Ghana's neglected chronic disease epidemic: a developmental challenge.. Ghana Med J.

[19] Browne EN, Frimpong E, Sievertsen J, Hagen J, Hamelmann C (2000). Malariometric update for the rainforest and savanna of Ashanti region, Ghana.. Ann Trop Med Parasitol.

[20] Krefis AC, Schwarz NG, Nkrumah B, Acquah S, Loag W (2010). Principal component analysis of socioeconomic factors and their association with malaria in children from the Ashanti Region, Ghana.. Malar J.

[21] Trape JF (1985). Rapid evaluation of malaria parasite density and standardization of thick smear examination for epidemiological investigations.. Trans R Soc Trop Med Hyg.

[22] Ghana Statistical Service (2010). http://www.statsghana.gov.gh/surveys/CENSUS2000/survey0/index.html.

[23] Marks F, Adu-Sarkodie Y, Hünger F, Sarpong N, Ekuban S (2010). Typhoid fever among children, Ghana.. Emerg Infect Dis.

[24] Pettorelli N, Vik JO, Mysterud A, Gaillard JM, Tucker CT (2005). Using the satellite-derivered NDVI to assess ecological responses to environmental change.. Trends in Ecology and Evolution.

[25] Haralick RM, Shanmugan K, Dinstein I (1973). Texture features for image classification. IEEE Trans.. Systems, Man and Cybernetics.

[26] de Jong SM, van der Meer FD (2004). Remote Sensing Image Analysis: Including the Spatial Domain.

[27] Russell PF, Santiago D (1934). Flight range of the *funestus-minimus* subgroup of *Anopheles* in the Philippines. First experiment with stained mosquitoes.. Am J Trop Med.

[28] Russell PF, Santiago D (1934). Flight range of *Anopheles* in the Philippines. Second experiment with stained mosquitoes.. Am J Trop Med.

[29] HI-STAR: Health improvement using Space technology and Resources (2002). http://www.isunet.edu/index.php?option=com_content&task=view&id=226&Itemid=251.

[30] Kreuels B, Kobbe R, Adjei S, Kreuzberg C, von Reden C (2008). Spatial variation of malaria incidence in young children from a geographically homogeneous area with high endemicity.. J Infect Dis.

[31] WHO (1982). Manual on environmental management for mosquito control. Offset Publication Number 66.

[32] Information about all districts in Ghana (2010). http://www.ghanadistricts.com/districts/?news&r=2&_=18.

[33] Cabrera BD, Valeza F (1978). Distribution and density of mosquitoes in two endemic areas for bancroftian filariasis in Sorsogon, Philippines.. Southeast Asian J Trop Med Public Health.

[34] Anosike JC, Nwoke BE, Okere AN, Oku EE, Asor JE (2007). Epidemiology of tree-hole breeding mosquitoes in the tropical rainforest of Imo State, south-east Nigeria.. Ann Agric Environ Med.

[35] Hopkins GHE (1952). Mosquitoes of the Ethiopian Region I. Larval Bionomics of Mosquitoes and Taxonomy of Culicine Larvae. 2^nd^ ed. with notes by Mattingly PF.

[36] Kerr JA (1933). Studies on the abundance, distribution and feeding habits of some West African mosquitoes.. Bull Ent Res.

[37] Patz JA, Graczyk TK, Geller N, Vittor AY (2000). Effects of environmental change on emerging parasitic diseases.. Int J Parasitol.

[38] Afrane YA, Zhou G, Lawson BW, Githeko AK, Yan G (2007). Life-table analysis of Anopheles arabiensis in western Kenya highlands: effects of land covers on larval and adult survivorship.. Am J Trop Med Hyg.

[39] Krefis AC, Schwarz NG, Krüger A, Fobil J, Nkrumah B (2011). Modeling the Relationship between Precipitation and Malaria Incidence in Children from a Holoendemic Area in Ghana.. Am J Trop Med Hyg.

[40] Kalyani S, Swarup KS (2010). Supervised fuzzy C-means clustering technique for security assessment and classification in power systems.. IJEST.

[41] Wie W, Zhang X, Chen X, Tang J, Jiang M (2008). Wetland Mapping using Subpixel Analysis and Decision Tree Classification in the Yellow River Delta Area.. ISPRS.

[42] Lawrence R, Hurst R, Weaver T, Aspinall R (2006). Mapping Prairie Pothole Communities with Multitemporal Ikonos Satellite Imagery.. PE&RS.

[43] Brooker S, Clarke S, Njagi JK, Polack S, Mugo B (2004). Spatial clustering of malaria and associated risk factors during an epidemic in a highland area of western Kenya.. Trop Med Int Health.

[44] Leonardo LR, Rivera PT, Cristostomo BA, Sarol JN, Bantayan NC (2005). A study of the environmental determinants of malaria and schistosomiasis in the Philippines using Remote Sensing and Geographic Information Systems.. Parassitologi.

[45] Minakawa N, Sonye G, Mogi M, Githeko A, Yan G (2002). The effects of climatic factors on the distribution and abundance of malaria vectors in Kenya.. J Med Entomol.

[46] Warrel DA, Gilles HM (2002). Essential malariology. 4^th^ ed.

[47] Uguru NP, Onwujekwe OE, Tasie NG, Uzochukwu BS, Ezeoke UE (2010). Do consumers' preferences for improved provision of malaria treatment services differ by their socio-economic status and geographic location? A study in southeast Nigeria.. BMC Public Health.

[48] Peterson I, Borrell LN, El-Sadr W, Teklehaimanot A (2009). Individual and household level factors associated with malaria incidence in a highland region of Ethiopia: a multilevel analysis.. Am J Trop Med Hyg.

