# Supplementary material for: Spatial Analysis of Land Cover Determinants of Malaria Incidence in the Ashanti Region, Ghana
Source: PLoS One. 2011 Mar 23;6(3):e17905. doi: 10.1371/journal.pone.0017905 (PMC3063166; doi:10.1371/journal.pone.0017905)
Supplement: Table S1 — Proportion (in %) of land cover around a 1.5 km village centre radius. Swampy area: either the presence of a river or stream nearby or near the ground agricultural crops (such as eggplants, maize, tomatoes, pepper). (DOC) [file pone.0017905.s001.doc]

| Village radius | Banana/Plantain | Cacao | Palm trees | Oranges | Deforested area and roads | Built-up areas (Houses) | Swampy area | Water | Forest |
| --- | --- | --- | --- | --- | --- | --- | --- | --- | --- |
| Agogo | 20.8 | 7.8 | 3.1 | 5.2 | 11.2 | 5.2 | 38.4 | 0.1 | 4.8 |
| Akutuase | 12.0 | 10.3 | 3.9 | 28.6 | 13.2 | 2.6 | 4.4 | 1.0 | 24.0 |
| Amantena | 11.1 | 24.2 | 24.7 | 21.8 | 2.8 | 0.8 | 5.3 | 0.1 | 8.5 |
| Domeabra | 14.3 | 23.8 | 16.8 | 18.2 | 9.3 | 5.5 | 7.0 | 0.0 | 4.8 |
| Hwidiem | 22.9 | 11.7 | 9.1 | 12.7 | 8.5 | 3.9 | 25.9 | 0.0 | 5.2 |
| Juansa | 13.3 | 25.1 | 16.2 | 19.3 | 9.7 | 5.8 | 7.2 | 0.0 | 3.2 |
| Kyekyebiase | 8.2 | 28.7 | 20.8 | 26.9 | 2.9 | 1.2 | 6.4 | 0.0 | 4.8 |
| Nyaboo | 22.6 | 14.4 | 10.2 | 2.3 | 25.8 | 7.1 | 11.0 | 0.0 | 3.3 |
| Obenimase | 17.6 | 26.0 | 12.1 | 3.7 | 19.5 | 2.2 | 6.2 | 0.6 | 10.1 |
| Patriensah | 23.8 | 17.1 | 10.2 | 1.9 | 23.7 | 5.7 | 11.3 | 0.0 | 1.6 |
| Pekyerekye | 3.9 | 30.8 | 19.8 | 13.1 | 11.6 | 1.3 | 8.5 | 3.7 | 7.2 |
| Wioso | 11.6 | 14.6 | 4.6 | 25.9 | 9.1 | 1.2 | 0.1 | 1.1 | 28.7 |
