# Supplementary material for: Spatial Analysis of Land Cover Determinants of Malaria Incidence in the Ashanti Region, Ghana
Source: PLoS One. 2011 Mar 23;6(3):e17905. doi: 10.1371/journal.pone.0017905 (PMC3063166; doi:10.1371/journal.pone.0017905)
Supplement: Table S2 — Proportion (in %) of land cover around a 1 km village centre radius. Swampy area: either the presence of a river or stream nearby or near the ground agricultural crops (such as eggplants, maize, tomatoes, pepper). (DOC) [file pone.0017905.s002.doc]

| Village radius | Banana/Plantain | Cacao | Palm trees | Oranges | Deforested area and roads | Built-up areas (Houses) | Swampy area | Water | Forest |
| --- | --- | --- | --- | --- | --- | --- | --- | --- | --- |
| Agogo | 21.7 | 6.5 | 2.0 | 3.7 | 23.3 | 7.0 | 36.5 | 0.1 | 3.6 |
| Akutuase | 12.7 | 9.1 | 3.4 | 23.8 | 18.2 | 4.9 | 4.5 | 1.0 | 22.3 |
| Amantena | 12.2 | 25.4 | 22.1 | 20.1 | 4.5 | 1.2 | 5.6 | 0.2 | 7.2 |
| Domeabra | 19.0 | 22.3 | 11.0 | 14.6 | 13.7 | 7.8 | 8.6 | 0.0 | 2.7 |
| Hwidiem | 24.8 | 12.4 | 7.0 | 12.8 | 11.9 | 6.2 | 20.6 | 0.0 | 4.2 |
| Juansa | 18.0 | 22.2 | 12.0 | 14.9 | 13.2 | 8.4 | 8.8 | 0.0 | 2.2 |
| Kyekyebiase | 11.7 | 28.5 | 15.6 | 26.1 | 5.9 | 2.6 | 6.1 | 0.0 | 3.3 |
| Nyaboo | 25.6 | 15.9 | 9.1 | 1.9 | 25.8 | 7.2 | 12.7 | 0.0 | 0.9 |
| Obenimase | 21.0 | 28.3 | 7.7 | 4.7 | 22.2 | 3.4 | 6.0 | 0.2 | 4.0 |
| Patriensah | 26.1 | 14.2 | 4.9 | 0.9 | 31.6 | 8.5 | 8.7 | 0.0 | 0.9 |
| Pekyerekye | 3.4 | 31.6 | 17.0 | 13.2 | 15.3 | 2.3 | 8.5 | 3.7 | 4.8 |
| Wioso | 11.2 | 4.3 | 4.3 | 25.3 | 12.1 | 2.3 | 2.8 | 0.7 | 26.3 |
