# Supplementary material for: Spatial Analysis of Land Cover Determinants of Malaria Incidence in the Ashanti Region, Ghana
Source: PLoS One. 2011 Mar 23;6(3):e17905. doi: 10.1371/journal.pone.0017905 (PMC3063166; doi:10.1371/journal.pone.0017905)
Supplement: Table S3 — Proportion (in %) of land cover around a 0.5 km village centre radius. Swampy area: either the presence of a river or stream nearby or near the ground agricultural crops (such as eggplants, maize, tomatoes, pepper). (DOC) [file pone.0017905.s003.doc]

| Village radius | Banana/Plantain | Cacao | Palm trees | Oranges | Deforested area and roads | Built-up areas (Houses) | Swampy area | Water | Forest |
| --- | --- | --- | --- | --- | --- | --- | --- | --- | --- |
| Agogo | 22.4 | 4.5 | 1.0 | 2.1 | 18.2 | 10.0 | 31.8 | 0.1 | 2.9 |
| Akutuase | 14.4 | 7.1 | 1.7 | 17.8 | 28.1 | 11.4 | 6.5 | 0.6 | 12.4 |
| Amantena | 19.0 | 25.5 | 12.6 | 14.4 | 9.2 | 3.4 | 7.5 | 0.8 | 6.0 |
| Domeabra | 25.5 | 12.4 | 0.5 | 5.0 | 28.7 | 17.2 | 10.1 | 0.0 | 0.1 |
| Hwidiem | 29.5 | 12.8 | 0.4 | 7.7 | 19.4 | 16.0 | 7.7 | 0.0 | 3.9 |
| Juansa | 27.2 | 9.8 | 3.2 | 2.8 | 30.2 | 19.4 | 9.1 | 0.0 | 0.1 |
| Kyekyebiase | 19.6 | 24.5 | 1.0 | 16.1 | 17.2 | 7.9 | 7.0 | 0.0 | 1.8 |
| Nyaboo | 25.7 | 14.9 | 5.5 | 1.0 | 29.3 | 12.3 | 9.5 | 0.0 | 0.3 |
| Obenimase | 30.0 | 21.5 | 6.4 | 3.4 | 28.6 | 10.4 | 5.5 | 0.0 | 0.2 |
| Patriensah | 27.3 | 6.4 | 0.9 | 0.5 | 41.0 | 14.5 | 5.7 | 0.0 | 0.3 |
| Pekyerekye | 2.3 | 26.4 | 12.0 | 10.5 | 25.3 | 7.5 | 9.5 | 4.1 | 2.2 |
| Wioso | 12.6 | 11.3 | 2.0 | 19.2 | 26.9 | 8.1 | 3.7 | 0.4 | 15.7 |
