# Supplementary material for: Spatial Analysis of Land Cover Determinants of Malaria Incidence in the Ashanti Region, Ghana
Source: PLoS One. 2011 Mar 23;6(3):e17905. doi: 10.1371/journal.pone.0017905 (PMC3063166; doi:10.1371/journal.pone.0017905)
Supplement: Table S4 — Influence of determinants on malaria incidence. Association of land cover with malaria incidence using Poisson regression analysis in radii of 0.5 km, 1 km, 1.5 km, and 2 km around each village. Land cover proportions were analysed as continuous variables and were scaled by units as per 2% increase in radii coverage by built-up areas (houses), per increase in open water of 1%, per increase of deforested area and roads of 5%, per increase in forest, swampy area, banana/plantain, oranges, cacao, and palm tree vegetation of 10%, respectively, and human population density as per 1,000 inhabitants. (DOC) [file pone.0017905.s004.doc]

| Radius | 2 km | | | 1.5 km | | | 1 km | | | 0.5 km | | |
| --- | --- | --- | --- | --- | --- | --- | --- | --- | --- | --- | --- | --- |
| Determinant | RR | 95% CI | p-value | RR | 95% CI | p-value | RR | 95% CI | p-value | RR | 95% CI | p-value |
| Population density | 0.87 | 0.70 – 1.07 | 0.176 | 0.87 | 0.70 – 1.07 | 0.176 | 0.87 | 0.70 – 1.07 | 0.176 | 0.87 | 0.70 – 1.07 | 0.176 |
| Built-up areas (houses) | 2.24 | 1.54 – 3.24 | <0.001 | 1.32 | 0.89 – 1.97 | 0.171 | 1.22 | 0.87 – 1.76 | 0.238 | 0.92 | 0.78 – 1.08 | 0.309 |
| Deforested area and roads | 1.00 | 0.70 – 1.44 | 0.988 | 0.93 | 0.68 – 1.26 | 0.619 | 1.19 | 0.96 – 1.48 | 0.110 | 0.77 | 0.63 – 0.92 | 0.005 |
| Forest | 0.53 | 0.28 – 0.99 | 0.029 | 0.65 | 0.33 – 1.27 | 0.208 | 0.67 | 0.33 – 1.35 | 0.264 | 0.83 | 0.33 – 2.08 | 0.695 |
| Swampy area | 1.43 | 1.33 – 1.55 | <0.001 | 1.42 | 1.32 – 1.53 | <0.001 | 1.44 | 1.32 – 1.58 | <0.001 | 1.46 | 1.24 – 1.71 | <0.001 |
| Water | 0.70 | 0.37 – 1.32 | 0.270 | 0.69 | 0.35 – 1.34 | 0.274 | 0.71 | 0.36 – 1.40 | 0.323 | 0.79 | 0.44 - 1.40 | 0.418 |
| Banana/Plantain | 3.25 | 2.23 – 4.76 | <0.001 | 2.34 | 1.36 – 4.02 | 0.002 | 1.93 | 1.04 – 3.58 | 0.038 | 1.22 | 0.67 – 2.22 | 0.508 |
| Oranges | 0.63 | 0.44 – 0.91 | 0.012 | 0.60 | 0.43 – 0.84 | 0.003 | 0.58 | 0.41 – 0.84 | 0.004 | 0.50 | 0.27 – 0.94 | 0.032 |
| Cacao | 0.48 | 0.33 – 0.70 | <0.001 | 0.51 | 0.39 – 0.66 | <0.001 | 0.57 | 0.42 – 0.77 | <0.001 | 0.50 | 0.31 – 0.80 | 0.004 |
| Palm trees | 0.59 | 0.43 – 0.81 | <0.001 | 0.49 | 0.34 – 0.71 | <0.001 | 0.40 | 0.24 – 0.67 | <0.001 | 0.37 | 0.09 – 1.47 | 0.160 |
